# Nanoengineered Silica-Based Biomaterials for Regenerative Medicine

**DOI:** 10.3390/ijms25116125

**Published:** 2024-06-01

**Authors:** Mohamed A. A. Abdelhamid, Hazim O. Khalifa, Mi-Ran Ki, Seung Pil Pack

**Affiliations:** 1 Department of Biotechnology and Bioinformatics, Korea University, Sejong-Ro 2511, Sejong 30019, Republic of Korea; mohamed42@korea.ac.kr; 2 Department of Botany and Microbiology, Faculty of Science, Minia University, Minia 61519, Egypt; 3 Department of Veterinary Medicine, College of Agriculture and Veterinary Medicine, United Arab Emirates University, Al Ain P.O. Box 1555, United Arab Emirates; hazimkhalifa@uaeu.ac.ae; 4 Department of Pharmacology, Faculty of Veterinary Medicine, Kafrelsheikh University, Kafr El-Sheikh 33516, Egypt; 5 Institute of Industrial Technology, Korea University, Sejong-Ro 2511, Sejong 30019, Republic of Korea

**Keywords:** regenerative medicine, nanoengineered silica, biocompatibility, tissue engineering, targeted drug delivery, biomimetic scaffolds, stem cell therapy, biomedical imaging

## Abstract

The paradigm of regenerative medicine is undergoing a transformative shift with the emergence of nanoengineered silica-based biomaterials. Their unique confluence of biocompatibility, precisely tunable porosity, and the ability to modulate cellular behavior at the molecular level makes them highly desirable for diverse tissue repair and regeneration applications. Advancements in nanoengineered silica synthesis and functionalization techniques have yielded a new generation of versatile biomaterials with tailored functionalities for targeted drug delivery, biomimetic scaffolds, and integration with stem cell therapy. These functionalities hold the potential to optimize therapeutic efficacy, promote enhanced regeneration, and modulate stem cell behavior for improved regenerative outcomes. Furthermore, the unique properties of silica facilitate non-invasive diagnostics and treatment monitoring through advanced biomedical imaging techniques, enabling a more holistic approach to regenerative medicine. This review comprehensively examines the utilization of nanoengineered silica biomaterials for diverse applications in regenerative medicine. By critically appraising the fabrication and design strategies that govern engineered silica biomaterials, this review underscores their groundbreaking potential to bridge the gap between the vision of regenerative medicine and clinical reality.

## 1. Introduction

Regenerative medicine stands at the forefront of medical innovation, harnessing the body’s intrinsic regenerative potential to revolutionize healthcare [1]. This groundbreaking field endeavors to restore or replace damaged, diseased, or dysfunctional tissues and organs, offering immense promise for treating a wide spectrum of debilitating conditions [2,3]. However, translating this vision into a clinical reality necessitates overcoming significant hurdles [4]. Traditional therapeutic approaches often grapple with limitations in biocompatibility, a lack of precise control over cellular behavior, and difficulties in mimicking the intricate architecture of natural tissues [5]. These limitations hinder the ability to establish a microenvironment that effectively promotes cell adhesion, proliferation, differentiation, and organization into functional tissues.

Fortunately, advancements in nanotechnology have yielded a promising solution for tissue regeneration: a diverse range of nanomaterials for scaffold development. Regenerative medicine is exploring a range of these nanomaterials, including metal nanoparticles [6,7], polymers [8], carbon-based structures [9], ceramics [10], and hydrogels [11], each with unique properties for tissue repair. However, silica-based nanomaterials have emerged as particularly promising candidates due to their advantageous characteristics. Their inherent biocompatibility ensures excellent biotolerance within the body [12]. Furthermore, the readily tailorable surface chemistry of silica allows for the meticulous design of customized platforms that target specific functionalities critical for regenerative medicine applications. By engineering these materials at the nanoscale, researchers can meticulously manipulate their size, shape, and surface characteristics to interact with cells and biological processes in highly specific ways [13].

Silica-based biomaterials offer a multitude of functionalities within regenerative medicine. Notably, they serve as versatile platforms for drug delivery, enabling precise control over the release profiles of a broad spectrum of therapeutic agents. This capability facilitates the establishment of targeted and sustained drug delivery, potentially leading to heightened treatment efficacy with minimized side effects [14]. Furthermore, these materials play a significant role in tissue engineering. Their ability to mimic the native tissue microenvironment fosters cellular growth and differentiation, facilitating the fabrication of functional tissues for repair and regeneration [15]. Beyond these applications, the integration of bioactive silica nanoparticles with stem cell therapy holds exciting prospects [16]. By influencing stem cell behavior and enhancing their differentiation and regenerative potential, this approach has the potential to further expand the therapeutic landscape of regenerative medicine. Finally, silica-based materials boast tailored properties that make them valuable in biomedical imaging [17]. They can be functionalized to facilitate non-invasive diagnostics and treatment efficacy monitoring in regenerative medicine applications.

This review explores the exciting potential of engineered silica-based biomaterials in regenerative medicine. We will explore their applications in targeted drug delivery, bone regeneration, stem cell therapy, and biomedical imaging. We will focus on how these materials are designed to influence cell behavior, promote tissue growth, and enable real-time monitoring of the healing process. Examples include engineering silica nanoparticles to stimulate bone formation and designing scaffolds with optimal structures for cell growth. We will also explore how silica can be functionalized for imaging, allowing researchers to track progress. By examining recent research and ongoing challenges, this review aims to highlight the transformative potential of silica-based materials in the future of regenerative medicine.

## 2. Synthesis and Functionalization Approaches for Silica-Based Materials

### 2.1. Synthesis Approaches for Silica-Based Materials

Silica nanoparticles exhibit remarkably tunable porosity, enabling the creation of mesoporous materials with intricate internal channels and hollow structures. This results in a high surface-area-to-volume ratio, a crucial property for many applications [18]. This versatility is attributed to the presence of unbound silanol (Si-OH) groups on the silica surface. These groups not only contribute to the low density of the material but also facilitate the formation of hollow structures. This unique property allows for the creation of heterostructures and core–shell nanoparticles by combining silica with other materials, offering tailored properties for various applications.

Hydrothermal and sol–gel synthesis methods offer significant advantages over gas-phase techniques like flame hydrolysis and plasma for producing silica nanoparticles [19,20]. Compared to the harsh conditions and limited scalability of gas-phase methods, these approaches operate under milder temperatures and pressures, enhancing safety and reducing energy consumption. Additionally, hydrothermal and sol–gel methods are readily scalable for large-scale industrial production. Most importantly, these methods offer exquisite control over particle morphology, porosity, functionality, and surface area by allowing for the manipulation of various reaction parameters. This precise control enables the creation of tailored silica nanoparticles with the desired properties for specific applications. Furthermore, these methods can be adapted for continuous processing, potentially leading to cost reduction and improved production efficiency.

Both hydrothermal and sol–gel synthesis methods can also be tailored for sustainability. This can be achieved by utilizing less hazardous precursors and environmentally friendly solvents, minimizing their environmental impact compared to some gas-phase counterparts [21,22]. The core principle of these methods relies on the supersaturation and polymerization of silicic acid (Si(OH)_4_), leading to the formation of a gel network and, ultimately, the generation of discrete silica particles [23]. The properties of the final product are notably impacted by the acidity or basicity of the reaction medium [24]. Under acidic conditions, the enhanced flocculation and connectivity between silica particles promote gel formation. Conversely, basic conditions result in the production of individually dispersed nanoparticles due to the presence of a negative surface charge [25]. Additionally, the concentration of electrolytes plays a crucial role in shaping the morphology of the final silica particles. This interplay between reaction parameters and product characteristics underscores the versatility and control offered by hydrothermal and sol–gel synthesis methods.

Various techniques, such as microemulsion, precipitation, Stöber, and biomimetic syntheses, utilize different sets of conditions for silica gel or particle precipitation [18]. These methods are continuously being refined with the aim of ensuring economic and environmental sustainability in the production of silica nanoparticles. Furthermore, different-shaped silica materials, such as hexagonal, cubic, rod, and hollow structures, can be synthesized using these methods [26]. The use of templates, such as surfactants and block copolymers, plays a crucial role in directing the morphology of these materials [27]. By carefully selecting and adjusting the template and reaction conditions, it is possible to achieve a wide range of silica structures with specific properties tailored for various applications. Overall, hydrothermal and sol–gel synthesis methods offer a versatile and sustainable approach for the production of tailored silica nanoparticles, facilitating advancements in various fields.

#### 2.1.1. Microemulsion Approach for Silica Synthesis

**Figure 1:**
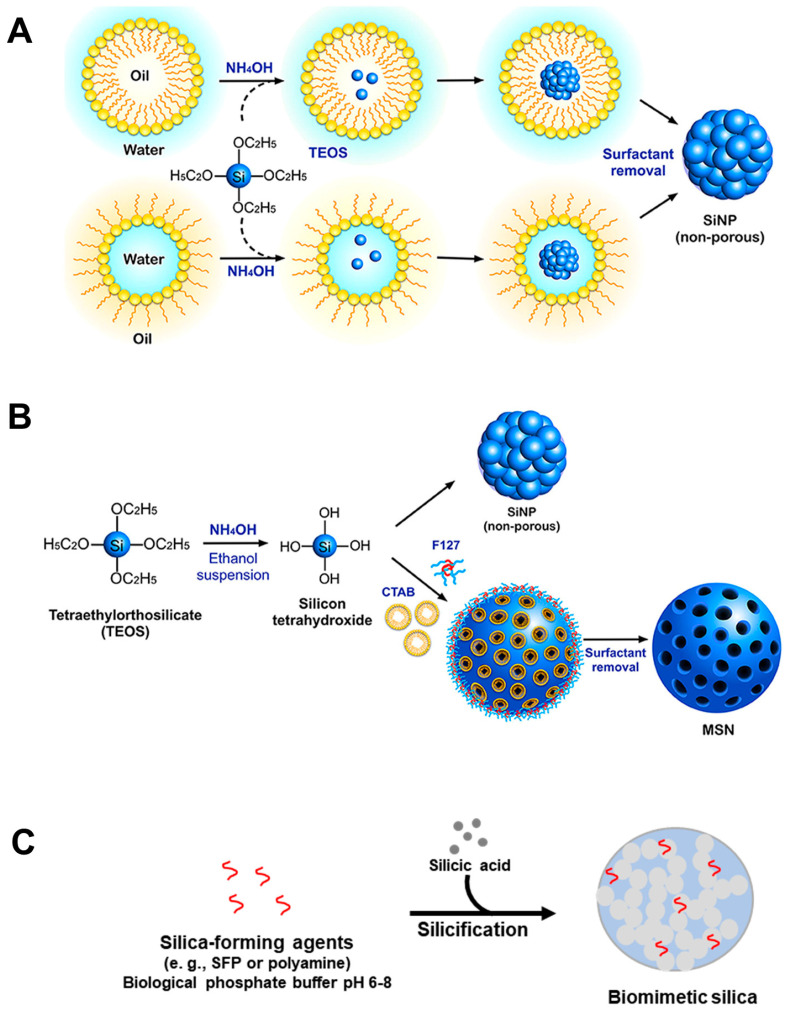
(**A**) Microemulsion, (**B**) Stöber, and (**C**) biomimetic approaches for silica nanoparticle synthesis. (F127: Pluronic F127 block copolymer surfactant; CTAB: cetyltrimethylammonium bromide). (**A**,**B**): Reproduced with permission from [33], copyright 2020 Frontiers (CC BY 4.0 DEED).

Microemulsion-based synthesis, particularly the water-in-oil approach, presents a unique platform for the synthesis of well-defined silica nanoparticles (Figure 1A). This technique leverages amphiphilic molecules, known as surfactants, to generate thermodynamically stable microemulsions, where aqueous nanodroplets are dispersed within a continuous oil phase [28,29]. These confined reaction vessels, termed nanoreactors, facilitate precise control over the nucleation and growth of silica nanoparticles, enabling the tailored manipulation of particle size, morphology, and size distribution with high fidelity [30]. A prominent advantage of the microemulsion route lies in its capability to generate significantly smaller silica particles compared to other solution-based synthesis methods. Furthermore, this approach enables the in situ incorporation of diverse functional moieties, such as metal cations, directly within the silica framework during the synthesis process [28]. This capability facilitates the surface modification of silica nanoparticles and the creation of multifunctional materials with tailored properties for diverse applications. This versatile method has been successfully employed to fabricate silica particles with a broad spectrum of sizes and characteristics, exemplifying its adaptability and effectiveness in the synthesis of diverse nanomaterials.

However, the microemulsion method presents inherent limitations. A significant consideration is the potential environmental impact associated with the utilization of organic solvents within the continuous oil phase [31]. Additionally, the confined environment of the microdroplets can present challenges in the efficient encapsulation and utilization of labile or sensitive molecules. Despite these limitations, ongoing research endeavors are continuously being directed towards the optimization of the microemulsion method for specific applications. A particularly intriguing area of exploration involves the application of this technique for the fabrication of core–shell nanoparticles, wherein a silica shell encapsulates a functional core material [32]. By addressing the environmental concerns and optimizing the entrapment efficacy, the microemulsion method holds immense potential for the controlled synthesis of advanced, functional silica nanoparticles tailored for diverse applications.

#### 2.1.2. Precipitation Approach for Silica Synthesis

The precipitation method dominates the global market for silica nanoparticles due to its cost-effectiveness and scalability [34]. This method achieves controlled precipitation by exceeding the solubility limit of a soluble silicate solution through acid–base neutralization [35]. Preheating the sodium silicate solution in water initiates the process, followed by the controlled addition of sulfuric acid and sodium sulfate to achieve the desired pH and prevent gel formation [18]. This crucial step significantly impacts the final product’s characteristics. The resulting precipitate undergoes rigorous purification and subsequent processing for various applications.

Several key reaction parameters, including pH, temperature, and reactant concentration, significantly influence the final product’s porosity and particle size [35]. pH dictates the surface charge of the particles, impacting their aggregation behavior and ultimately affecting particle size. Temperature governs the reaction kinetics and nucleation rate, influencing both particle size and morphology. Finally, reactant concentration determines the degree of supersaturation and can influence the particle size distribution and morphology [36,37].

While precipitated silica offers advantages in terms of lower energy consumption and production cost compared to other methods, it has limitations [38]. One challenge is the inherent trade-off between purity and cost, as precipitated silica often exhibits lower purity compared to other methods like pyrogenic synthesis. However, this drawback is often outweighed by the significant cost benefits. Another limitation is the limited control over particle properties like size and morphology. Ongoing research is exploring advanced processing techniques and alternative precursors to address these limitations and achieve greater control while maintaining the economic and scalability advantages that have established the precipitation method as a dominant player in the silica nanomaterial industry.

#### 2.1.3. Stöber Approach for Silica Synthesis

The Stöber method, first described by Stöber et al. in 1968, offers unparalleled control over the size, morphology, and surface functionalities of silica nanoparticles [39]. This method facilitates the synthesis of monodisperse spherical silica particles in the nano- to micrometer range (50–2000 nm) via the hydrolysis and condensation polymerization of alkoxysilanes under basic conditions (typically using ammonia) within a specific alcohol–water mixture (Figure 1B) [40]. Extensive research has elucidated the impact of various parameters, including the alcohol type, reactant concentrations, reaction temperature, and even the alkyl chain length of the alkoxysilanes, on the final particle characteristics [24]. Beyond enabling a broad spectrum of controllable particle diameters and size distributions, the Stöber method allows for the fabrication of microporous silica and the in situ functionalization of the silica surface during the synthesis process [41,42,43,44]. Additionally, successful adaptation of the method has been achieved by replacing ammonia with sodium hydroxide as a catalyst [45]. Recent advancements even involve the utilization of modified Stöber methods within continuous microfluidic reactors, facilitating efficient synthesis [46,47].

However, despite its versatility, the Stöber method encounters several challenges. The use of potentially hazardous chemicals like alkoxysilanes, the requirement for organic solvents, and the relatively high cost of alkoxysilane precursors hinder its widespread commercial application. While alternative methods like precipitation and microemulsion offer advantages in terms of cost-effectiveness and scalability, the Stöber method remains uniquely advantageous for applications demanding precise control over particle characteristics. This inherent trade-off between controllability and the limitations in environmental impact and cost necessitates careful consideration when selecting the most suitable method for specific needs.

#### 2.1.4. Biomimetic and Bioinspired Approaches for Silica Synthesis

Nature offers fascinating inspiration for crafting advanced materials [48]. Marine organisms, encompassing a diverse taxonomic group including diatoms and sponges, have captivated researchers in the field of biosilica synthesis due to their remarkable ability to biomineralize intricate siliceous microstructures [22,49]. The natural process by which these organisms form their intricate exoskeleton structures, known as biosilicification, holds immense potential for researchers seeking to mimic this process and create novel biomimetic materials [50]. By understanding the intricate biological mechanisms employed by these organisms, scientists hope to develop new materials with tailored properties for diverse applications, ranging from drug delivery and tissue engineering to photonics and advanced composites [51].

Diatoms, a prolific class of unicellular eukaryotic microalgae, are known for their remarkable ability to utilize silicon from their environment to construct their cell walls, known as frustules [52]. These frustules, essentially biogenic “glass houses” composed of hydrated amorphous silica, exhibit species-specific nano- to mesoporous architectures that captivate scientists with their exquisite beauty and captivating properties. For more than two centuries, the unique combination of biocompatibility, chemical inertness, mechanical robustness, high surface area, and distinct optical properties offered by diatom biosilica has captivated scientists and inspired innovation. This fascination has translated into a diverse array of real-world applications, including photonics, separation technology, biomedicine, sensing, and energy conversion and storage [53].

Unraveling the secrets behind diatom biosilica formation has been a key scientific pursuit in recent decades [54]. The biosynthesis of these remarkable structures occurs within specialized membrane-bound compartments called silica deposition vesicles (SDVs) [55]. Through intensified research efforts, scientists have been steadily elucidating the molecular mechanisms governing diatom silicification [56]. This research has revealed the crucial roles played by silicification-specific proteins, notably silaffins, which undergo specific post-translational modifications that significantly impact the structure and kinetics of silica formation [57]. Additionally, the identification of novel SDV membrane proteins like silicanin [58], tpSAP1, tpSAP2, and tpSAP3 [59], along with the discovery of lysine-rich motifs within silaffins [60], has significantly enhanced our understanding of diatom biosilica formation and its potential for diverse applications. These molecular insights provide a robust foundation for harnessing the unique properties of diatom biosilica and developing groundbreaking practical applications across various fields [61,62,63,64].

Similar to diatoms, siliceous sponges belonging to classes like Hexactinellida and Demospongiae also utilize biosilicification to construct their skeletal spicules, primarily composed of hydrated amorphous silica [22]. Within the biosilicification process, research initially identified silicatein, an enzyme embedded in specific demosponges such as *Tethya aurantia*, as the first protein implicated in this pathway. Silicatein exhibits structural similarities to cathepsin L, a cysteine protease enzyme, but contains a unique serine–histidine–asparagine triad instead of the characteristic cysteine active site [65]. While earlier studies suggested silicatein’s catalytic ability to condense silica precursors under mild conditions, more recent research has challenged its role as the primary enzyme, suggesting a possible alternative mechanism involving surface- or structure-directed biosilicification [66]. Additionally, the discovery of glassin, another protein identified in the skeleton of *Euplectella aspergillum*, contributes to our understanding of sponge biosilicification. However, the precise mechanism of glassin’s involvement requires further investigation [67].

The pursuit of biomimetic approaches for silica synthesis has emerged as a significant area of research due to their promising potential in various biotechnological applications [22,68]. These strategies deviate from traditional sol–gel methods by replicating biological conditions, using near-neutral pH and non-toxic solutions (Figure 1C). To circumvent the challenges of extracting native biomolecules (like zwitterionic silaffins and cationic long-chain polyamines) crucial for biogenic silica formation, researchers employ synthetic biomimetic analogs. These synthetic molecules meticulously mimic their natural counterparts, facilitating controlled silica synthesis under mild conditions, opening doors for innovative applications in biotechnology.

Researchers have explored various biomimetic approaches for silica synthesis, each offering distinct capabilities and limitations. One such approach utilizes externally applied forces to manipulate silaffin-derived R5 peptide, resulting in a diverse array of silica morphologies. This technique has successfully yielded novel structures, including arc-shaped structures, fiber-like silica, and sheet-like formations [69,70]. However, the employed experimental conditions, which may not be environmentally friendly, could potentially restrict the practical application of this method, particularly when dealing with sensitive biomolecules.

Several novel silica-forming peptides (SFPs) have been identified and reported for in vitro biomimetic silica synthesis. These peptides, including EctP1, EctP2, Salp1, and Volp1, typically initiate silica precipitation under mild conditions with ambient temperature and neutral pH (ranging from 6 to 8) in a phosphate buffer environment [71,72]. Recently, a synthetic peptide, RSGH, has been designed to facilitate biosilica synthesis under acidic conditions, expanding the versatility of this approach [73]. A recent study explored the manipulation of local reaction conditions to precisely control silica precipitation induced by the cationic peptide, SiNP-5 [74]. By varying factors like shaking intensity and the concentrations of both the peptide and precursor materials, the study achieved precise control over the formation of silica structures under mild conditions.

Self-assembling polypeptides are emerging as potent tools for biomimetic silica synthesis. These intricately designed molecules act as sophisticated biotemplates, dictating the controlled precipitation of silica through the precise arrangement of amino acids within their primary structure. This innovative approach, primarily utilizing amphiphilic lysine–leucine (LK) peptides, has yielded silica with a remarkable diversity of morphologies, including nanospheres, elongated nanowires, and nanorods [75]. Notably, even subtle modifications, such as peptide acetylation, have demonstrably influenced the final structure of the precipitated silica [76]. Further exploration into the influence of peptide properties on silica structure revealed the potential of peptide amphiphilicity as a control parameter. In this approach, ultrashort isoleucine–lysine peptides self-assembled into diverse structures, acting as templates for the controlled precipitation of silica with corresponding morphologies [77]. Additionally, research has identified amyloid protein fibrils as effective templates for the synthesis of core–shell silica filament structures with enhanced mechanical properties [78].

Building upon the success of self-assembling polypeptides, researchers have delved into further exploration of biomacromolecules for biomimetic silica synthesis. These studies have employed fully ordered biomacromolecules as dual-functioning catalysts and templates. Examples include proteins like lysozyme [79] and bovine serum albumin [80]. Furthermore, recent research has explored the genetic fusion of peptides with proteins such as ferritin or elastin-like polypeptide micelles to control the generation of sub-micron monodisperse silica nanoparticles [81,82]. These diverse strategies offer valuable insights into the intricate world of biomimetic silica synthesis. Each method presents unique advantages and considerations for potential applications, highlighting the expansive toolkit available for researchers in this exciting field.

#### 2.1.5. Hydrothermal Approach for Silica Synthesis

Hydrothermal synthesis stands out as a powerful method for crafting silica-based micro- and nanomaterials, especially those with well-defined pores [83]. It utilizes a sealed reaction vessel under elevated temperatures (typically ranging from 100 to 200 °C) and pressures, creating a precisely controlled environment that promotes highly ordered, crystalline structures with superior hydrothermal stability and mechanical properties compared to ambient methods [84]. Furthermore, this technique offers exceptional control over the final product’s porosity through meticulous adjustment of reaction parameters, enabling tailored pore sizes and distributions for specific functionalities [85]. While hydrothermal synthesis boasts faster reaction rates than conventional sol–gel processing, the high temperatures and pressures can lead to increased energy consumption. However, this technique remains a valuable tool for the targeted synthesis of high-quality silica nanoparticles with precisely tailored properties for diverse applications in catalysis, drug delivery, and separation technologies.

### 2.2. Functionalization Approaches for Silica Surfaces

**Figure 2:**
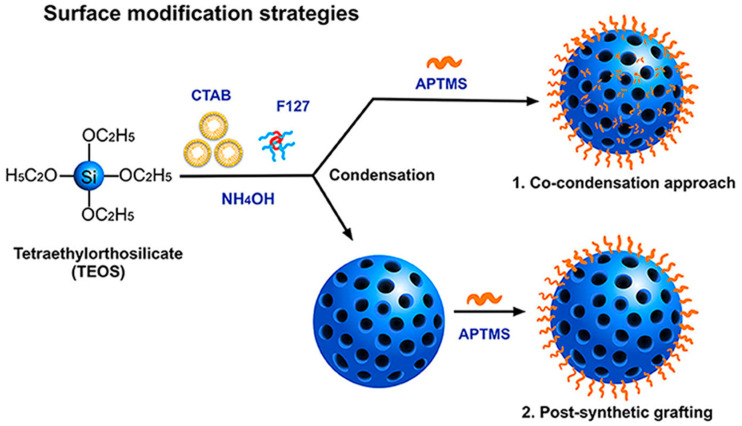
Surface modification strategies for silica nanoparticles. (F127: Pluronic F127 block copolymer surfactant; CTAB: cetyltrimethylammonium bromide; APTMS: [3-aminopropyl]trimethoxysilane). Reproduced with permission from [33], copyright 2020 Frontiers (CC BY 4.0 DEED).

Surface engineering is a critical process that unlocks the full potential of silica nanoparticles for diverse scientific and technological applications. This meticulous approach involves the strategic modification of the nanoparticle surface to introduce desired functionalities. Beyond mere decoration, surface engineering empowers researchers to create versatile platforms tailored for specific applications. Through this process, researchers can precisely control crucial properties of silica nanoparticles, including surface charge, hydrophilicity, biocompatibility, and targeting capabilities [86]. Various techniques are employed to impart desired functionalities to silica nanoparticles (Figure 2). The co-condensation approach directly introduces functionalities into the silica framework during sol–gel synthesis, utilizing organosilanes alongside starting materials [87]. Post-synthetic grafting extends the versatility, enabling the use of a broader range of functional groups, even those sensitive to harsh conditions [88]. Bifunctional coupling agents containing silane groups bridge the desired functional group with the silica nanoparticle surface [89]. Surface polymerization enhances the properties of silica nanoparticles by increasing surface functional group density, achieved through “grafting to” or “grafting from” approaches, providing precise control over functionality and density.

Additionally, emerging techniques like Janus nanoparticles and electrochemical deposition offer exciting possibilities. Janus nanoparticles, with distinct functionalities on opposing hemispheres, can be crafted using microfluidic techniques, enabling targeted interactions and controlled release properties [90]. Electrochemical deposition allows for the precise layer-by-layer assembly of functional molecules onto the silica nanoparticle surface, offering excellent control over surface chemistry and functionality [91,92,93]. Furthermore, click chemistry and bioconjugation introduce additional dimensions to silica nanoparticles functionalization, relying on highly efficient and specific reactions for rapid and selective surface modification [94,95]. These techniques enable the attachment of biomolecules like antibodies or enzymes to the surface of silica nanoparticles, expanding applications in biomedicine and biosensing. 

Notably, surface engineering plays a crucial role in tailoring silica nanoparticle biocompatibility. Unmodified silica nanoparticles can interact with and potentially damage biological molecules and are rapidly cleared from circulation. Surface modification techniques like PEGylation and liposome coating address these limitations by significantly reducing cytotoxicity and extending circulation time, making silica nanoparticles more suitable for biomedicine and drug delivery applications [96,97]. These diverse surface engineering strategies empower scientists to create versatile platforms for a wide range of applications, ultimately unlocking the true potential of silica materials.

## 3. Application of Engineered Silica in Regenerative Medicine

Engineered silica-based materials are emerging as a powerful platform for advancements in regenerative medicine (Table 1). Their inherent biocompatibility, precisely tunable porosity, and versatile surface functionalization capabilities make them ideally suited for controlled drug delivery, targeted therapeutic interventions, and the development of biomimetic scaffolds. This section delves into the diverse applications of engineered silica within regenerative medicine, showcasing their remarkable potential to revolutionize treatment efficacy and enhance patient outcomes in critical areas such as bone regeneration, stem cell therapy, and a broad spectrum of biomedical applications.

### 3.1. Engineered Silica-Based Materials for Drug Delivery

Silica-based biomaterials have emerged as a prominent class of nanocarriers in drug delivery systems (DDS) due to their unique properties and versatility. Their ability to encapsulate a diverse range of therapeutic agents with controlled release profiles makes them particularly attractive for applications in regenerative medicine. This section will explore the various aspects of how nanoengineered silica-based biomaterials serve as promising platforms for controlled and targeted drug delivery, ultimately contributing to advancements in regenerative medicine (Table 1).

**Figure 3:**
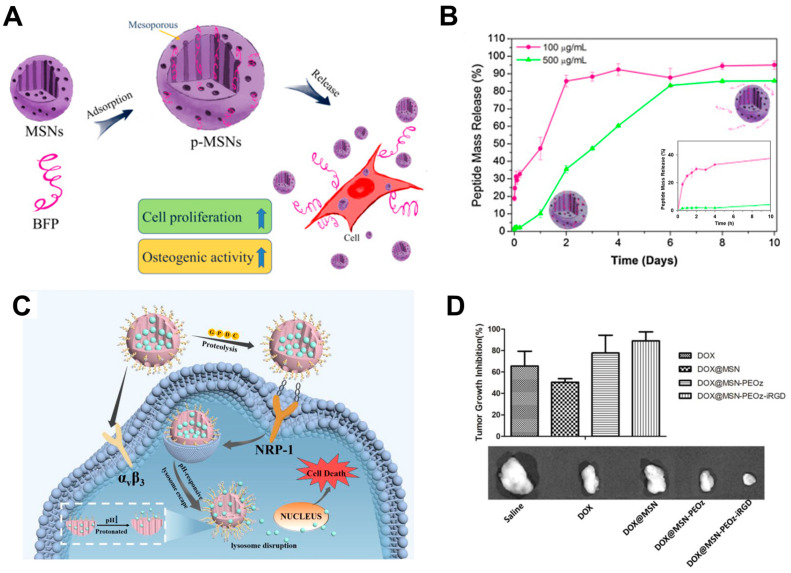
Mesoporous silica nanoparticles for targeted drug delivery. (**A**) Fabrication and in vitro cellular interactions of peptide-loaded MSNs (p-MSNs). (**B**) BFP release kinetics. (Inset: 10 h magnification). (**C**) Schematic depicting targeted delivery, penetration, and triggered release of PEOz-iRGD-modified MSNs in tumor cells. (**D**) Evaluation of tumor growth inhibition post-intravenous injection. (**A**,**B**): Reproduced with permission from [98], copyright 2015 Elsevier B.V. (**C**,**D**): Reproduced with permission from [99], copyright 2024 Elsevier B.V (CC BY-NC-ND 4.0 DEED).

Silica–polypeptide conjugates represent a versatile platform with the potential to revolutionize drug delivery in various areas of regenerative medicine. Their unique combination of biocompatibility, targeting specificity, and controlled release capabilities opens doors for advancements in bone regeneration, cancer therapy, and beyond. As research progresses, further exploration and refinement of these platforms hold immense promise for improving patient outcomes and advancing the field of regenerative medicine. A recent study by Luo et al. exemplifies the potential of these conjugates. Their work explored peptide-laden mesoporous silica nanoparticles (p-MSNs) for bone tissue engineering (Figure 3A,B) [98]. These p-MSNs incorporate a bone morphogenetic protein-7 (BMP-7)-derived peptide (BFP), functioning as controlled-release carriers for osteogenic factors. The study demonstrated successful encapsulation of the peptide within the p-MSN’s mesopores, while maintaining its structural integrity. Compared to bare nanoparticles, p-MSNs exhibited superior biocompatibility and cytocompatibility, as evidenced by increased cell proliferation, spreading, and alkaline phosphatase (ALP) activity in MG-63 cells. Furthermore, p-MSNs effectively stimulated the differentiation of human mesenchymal stem cells (hMSCs) into bone cells (osteogenesis), highlighting their potential to promote bone formation. These findings strongly suggest that p-MSNs hold promise as a biocompatible and effective material for applications in bone repair, regeneration, and bio-implant coatings.

**Table 1:** Emerging applications of nanoengineered silica in regenerative medicine.

Materials	Description	Functionalization	Advantages	Applications	References
Peptide-laden MSN	Slow-release system for osteogenic factor delivery	Bone-forming peptide (BFP) derived from BMP-7	Enhanced osteogenic differentiation of hMSCs (at BFP concenration of at least 500 μg/mL), good in vitro cytocompatibility, sustained BFP release	Drug delivery, bone repair, bone regeneration, bio-implant coatings	[98]
Dual-modified mesoporous silica nanoparticles	Drug delivery carrier for treating triple-negative breast cancer	iRGD peptide (targets αvβ3 integrin receptor) and pH-responsive PEOz polymer (facilitates lysosomal escape and drug release)	Selective targeting of cancer cells, deep tumor penetration, rapid intracellular drug release, reduced systemic toxicity	Drug delivery (cancer therapy)	[99]
Upconverting nanoparticle core encapsulated in mesoporous silica shell	Multifunctional carrier for combination therapy of metastatic spinal tumors	Loaded with IDO-derived peptide vaccine (AL-9), photosensitizer molecules, and PD-L1 inhibitor	Simultaneous PDT and immune checkpoint blockade, enhanced immune response and T-cell infiltration, reduced progression of metastatic tumors	Drug delivery (cancer therapy)	[100]
P4 peptide/silica hybrid particles	Carrier for sustained delivery of osteoinductive P4 peptide	None (inherent property of P4 peptide)	1.5-fold increase in P4 delivery to MC3T3 E1 cells (over 250 h), potential for synergistic osteogenesis when combined with hydroxyapatite	Drug delivery (bone regeneration)	[101]
pH-responsive nano-carrier (P4-VP@MCM-41)	MCM-41 as the container for controlled release of methotroxate drug	P4-VP as pH-sensitive gatekeepers	Controlled drug release (68.5% at pH 5 and 17% at pH 7 over 12 h), potentially higher drug concentration at tumor, reduced systemic exposure to the drug	Drug delivery (cancer therapy)	[102]
MSN-embedded core–shell nanofiber membrane	Scaffold for controlled delivery of growth factor (rhBMP-2) and antibiotic (gentamicin) for bone regeneration	Coaxial electrospinning: MSNs in the core, PVA and PCL polymers in the shell	Sustained growth factor release, enhanced bone regeneration, antibacterial properties, improved bioactivity, controlled drug release	Drug delivery (growth factors and antibiotics) and tissue engineering (periodontal tissue regeneration)	[103]
Silica-entrapped BMP2	Carrier system for BMP2 delivery	Encapsulation of BMP2	Increased BMP2 loading (72%), sustained release, enhanced bone formation at lower doses, improved BMP2 stability	Tissue engineering (bone regeneration)	[104]
GelMA/silanated silica composite	3D-printable scaffold for bone regeneration	Silanized silica particles embedded in GelMA	Improved printability, enhanced mechanical strength, promotes bone cell growth and differentiation	Tissue engineering (hard tissue regeneration)	[105]
Biosilica micropatterns on silk hydrogel	Micropatterned biocompatible surface for cell engineering	Inkjet printing of R5 peptide for silica biomineralization	High-resolution micropatterning, promoted cell alignment (hMSCs), avoids harsh chemicals	Tissue engineering (cell alignment)	[106]
Poly(MMA-co-TMSPMA)-star-SiO_2_ hybrid	3D-printable biomaterial ink for bone substitutes	Combination of star polymer and silica	Printable with tunable pores, mimics bone mechanics, promotes bone and blood vessel formation, supports pro-healing immune response	Tissue engineering (bone regeneration)	[107]
Biosilicified coccolithophore-derived coccoliths	Marine-inspired biomineral complex for bone graft substitute	Bioengineered coccoliths with MAP-EctP1 fusion protein for silica deposition	improved bone-forming minerals and promoted bone cell activity, supporting new bone formation	Tissue engineering (bone regeneration)	[108]
Bioactive SiO_2_ NPs	Carriers for promoting bone cell differentiation	Post-synthesis functionalization with calcium and phosphate ions	Small size (100 nm) for cellular uptake, promotes bone cell growth and differentiation, potentially single-dose treatment, good biocompatibility	Tissue engineering (bone regeneration) and potential stem cell therapy	[109]
HSA-coated MSN	Material for xenogenic-free stem cell culture	Surface modification with HSA protein	Improved stability in serum-free medium, enhanced stem cell uptake (80% of MSN), maintained differentiation potential	Stem cell therapy and tissue engineering	[16]
Bioinspired silica backpack on hASCs	Protective shell for stem cells	APTES for potential further functionalization	Enhanced survival in suspension and platform for future modifications (targeted therapy/differentiation)	Stem cell therapy and tissue engineering	[110]
3D-printed composite scaffolds (silica/PTHF/PCL)	Scaffolds for directing stem cell differentiation towards cartilage formation	Combination of silica with PTHF and PCL (composite functionalization)	Tunable channels for chondrogenic differentiation, improved scaffold properties (strength and biocompatibility)	Stem cell therapy and tissue engineering (cartilage regeneration)	[111]
Biotinylated dual-color fluorescent SiO_2_ NPs	Nanoparticles for bioimaging	Biotinylation for stability and potential targeting, dual fluorescent dyes (Oregon Green 488 and ATTO 647 N)	Improved stability, multicolor imaging, defined structure	Biomedical imaging (fluorescence optical nanoscopy)	[112]
Gd^3+^-loaded red fluorescent MSNs	Dual-mode probes for bioimaging	AIE dye for red fluorescence (reduced autofluorescence), Gd³⁺ for T1-weighted MRI contrast, APTES surface for potential bioconjugation	Dual-modality imaging (fluorescence and MRI) for comprehensive information, deep tissue penetration with red fluorescence, good biocompatibility (further in vivo studies needed)	Biomedical imaging (fluorescence microscopy, contrast-enhanced MRI)	[113]
YVO_4_:Eu^3+^@silica-NH-GDA-IgG bio-nanocomplexes	Ultra-small (20–25 nm) multifunctional spherical nanoparticles	YVO_4_:Eu^3+^ core (red emission), silica coating, NH-GDA linker for IgG attachment, MCF-7 specific antibodies	Enhanced red emission, biocompatible, targets MCF-7 cells via antibodies	Biomedical imaging (cancer cell labeling)	[114]
Mesoporous silica rods	Multifunctional MRI contrast agents	Maghemite nanocrystals, fluorophores (fluorescamine and Cyanine5) for potential multimodal imaging	High surface area, T2-weighted MRI contrast, potential for multimodal imaging	Biomedical imaging (T2-weighted MRI)	[115]
FMSN-MnO_2_-BCQ nanoparticles	Multimodal theranostic platform for tumor imaging and therapy	Degradable BSA-modified FMSN core, loaded with MnO_2_ (MRI contrast, Fenton reaction), BCQ (NIR-II FL, CDT)	Dual-modality imaging (MRI, NIR-II FL), degradable, TME-responsive drug release, self-reinforcing CDT	Biomedical imaging (MRI, NIR-II FL imaging)	[116]
Multifunctional theranostic nanoparticles (MDNs)	Bimodal imaging and combination therapy platform	Doxorubicin-loaded MSN core (drug delivery, biodegradable), sub-6 nm CuS nanodots shell (PET, photothermal therapy, renal clearance).	High tumor uptake, bimodal imaging (PET, photoacoustic), triggered drug release, biodegradable and renally clearable	Biomedical imaging (PET, photoacoustic imaging) and cancer therapy (chemotherapy, photothermal therapy)	[117]

Abbreviations: hMSCs, human mesenchymal stem cells; hASCs, human adipose-derived mesenchymal stem cells; BMP-7, bone morphogenetic protein-7; PEOz, poly(2-ethyl-2-oxazoline); iRGD, tumor-homing cyclic peptide (CRGDKGPDC); IDO, indoleamine 2,3-dioxygenase; PD-L1, programmed cell death ligand 1; P4-VP, Poly(4-vinylpyridine); MCM-41, mesoporous silica nanoparticles; MSN, mesoporous silica nanoparticle; PVA, Poly(vinyl alcohol); PCL, Poly(caprolactone); BMP2, bone morphogenetic protein 2; GelMA, gelatin methacrylate; Poly(MMA-co-TMSPMA, poly(methyl methacrylate-*co*-3-(trimethoxysilyl)propyl methacrylate); MAP, mussel adhesive protein; EctP1, silica-forming peptide; HSA, human serum albumin; APTES, (3-Aminopropyl)triethoxysilane; PTHF, poly(tetrahydrofuran); SiO_2_, silica; NPs, nanoparticles; AIE, aggregation-induced emission; MRI, magnetic resonance imaging; FMSN, fusiform-like mesoporous silica nanoparticles; CDT, chemodynamic therapy; BSA, bovine serum albumin; YVO_4_, yttrium(III) orthovanadate; PDT, photodynamic therapy; rhBMP-2, recombinant bone morphology protein-2; GDA, glutaraldehyde; BCQ, BSA and NIR-II small molecule (CQ4T); PET, positron emission tomography.

Another study explored a novel peptide–silica conjugate for targeted drug delivery in cancer treatment. The conjugate, derived from mesoporous silica, was dually modified with a tumor-homing cyclic peptide iRGD and a pH-responsive polymer PEOz (Figure 3C,D) [99]. The study demonstrated that the conjugate was selectively bound to breast cancer cells and penetrated deeply into tumor tissue. It exhibited enhanced cytotoxicity (killing of cancer cells) and efficient escape from lysosomes (cellular compartments that degrade materials). In a mouse model, the conjugate significantly inhibited tumor growth without causing side effects. This innovative approach presents a promising strategy for overcoming challenges in nanocarrier penetration and drug release, potentially leading to improved cancer therapy.

**Figure 4:**
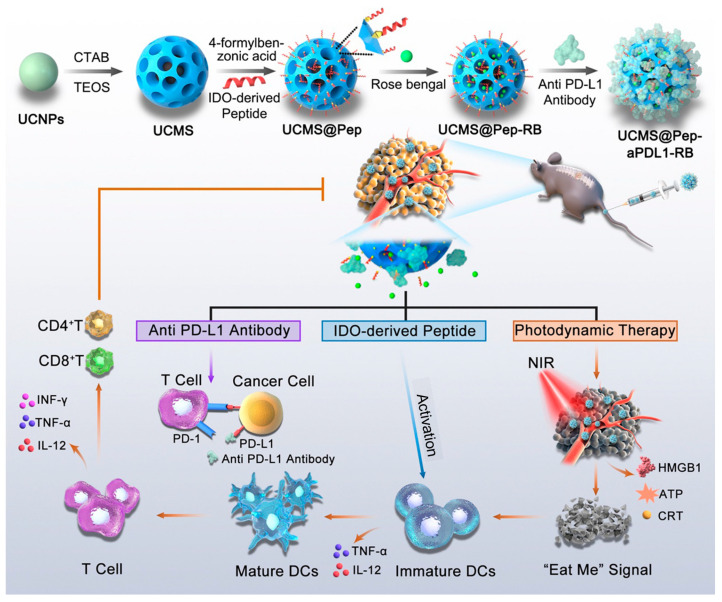
Targeted drug delivery for spinal tumor therapy. Schematic of a nanocarrier delivering a combo therapy (photosensitizer, peptide vaccine, PD-L1 inhibitor) for spinal tumors via PDT and immune stimulation. (UCMS: Upconverting nanoparticle core encapsulated in mesoporous silica shell). Reproduced with permission from [100], copyright 2021 Springer Nature (CC BY 4.0 DEED).

A sophisticated nanocarrier system targeted drug delivery in cancer immunotherapy was developed, utilizing an upconverting nanoparticle core encapsulated within a mesoporous silica shell (UCMS) (Figure 4) [100]. This novel UCMS platform was then ingeniously loaded with a potent therapeutic cocktail, encompassing a photosensitizer, an IDO-derived peptide vaccine (AL-9), and a PD-L1 inhibitor. The IDO-derived peptide vaccine was delivered to stimulate immune response via dendritic cell recognition, while the PD-L1 inhibitor was incorporated to enhance cytotoxic T-cell activity. Furthermore, near-infrared-activated photodynamic therapy (PDT) was facilitated by the UCMS platform, inducing immunogenic cell death (ICD) and fostering effector T-cell infiltration. This synergistic combination of PDT-induced ICD, peptide vaccine, and immune checkpoint blockade, delivered via the UCMS@Pep-aPDL1 construct, demonstrated significant potential in augmenting local and systemic antitumor immunity. Consequently, the progression of metastatic tumors was mitigated. This pioneering approach underscores the promising role of engineered silica-based materials, specifically the UCMS platform, in facilitating targeted drug delivery strategies for combating metastatic cancers.

Another study unveiled a novel strategy for delivering bone-promoting signals using biomimetic silica [101]. This method harnesses a protein fragment, P4, derived from bone morphogenetic protein (BMP), a crucial molecule for bone formation. P4 was incorporated into silica nanoparticles, creating sustained-release carriers for the bone-growth signal. Interestingly, the silica shell not only protected P4 but also enhanced its delivery to bone-forming cells, potentially amplifying its effectiveness. Furthermore, the study took the approach a step further. Researchers anchored P4-loaded silica onto a bone-mimicking material. This biomimetic co-delivery system demonstrated superior bone-growth potential compared to using silica or P4 alone. This approach provides a promising avenue for controlled release of bone-growth signals and holds significant potential for applications in regenerative medicine, ultimately aiding bone healing and regeneration.

Silica–biopolymer conjugates hold immense promise for regenerative medicine by revolutionizing drug delivery in this rapidly evolving field. These innovative platforms leverage the synergistic combination of silica nanoparticles and biopolymers. Silica nanoparticles offer inherent biocompatibility and tunable porosity, allowing for the encapsulation of therapeutic agents within their mesopores. Biopolymers, on the other hand, contribute targeting specificity and versatility to the system. One prominent example of these conjugates was a chitosan-grafted mesoporous silica material (MSM), designed to address the challenge of poor water solubility faced by many promising drugs [118]. This material incorporated chitosan, a biocompatible polymer, grafted onto the surface of mesoporous silica. This modification allowed for efficient loading of the drug within the silica carrier’s pores, significantly improving its dissolution rate and bioavailability. This research, encompassing both in vitro and in vivo investigations, demonstrated the successful application of MSMs in enhancing the oral delivery of poorly water-soluble drugs. Additionally, the study confirmed the biocompatibility and safety of MSMs, supporting their further exploration for potential application in various therapeutic areas.

**Figure 5:**
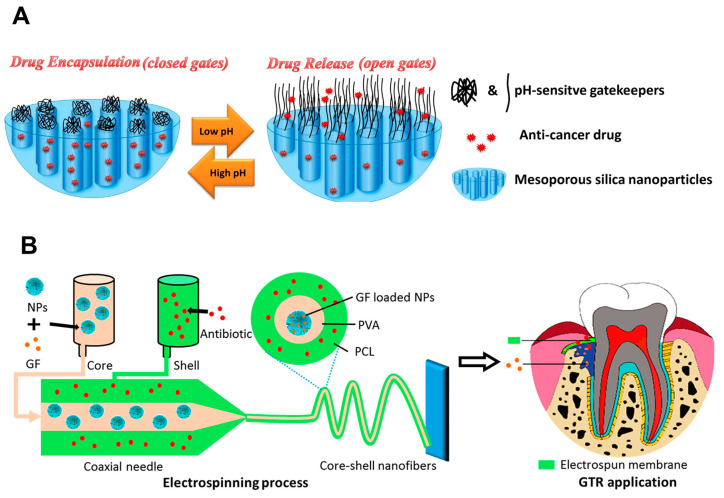
Biopolymer-enhanced drug delivery with silica nanoparticles. (**A**) pH-triggered drug release: a gatekeeper system controls drug release from silica based in acidic environments. (**B**) Dual drug delivery for bone regeneration: core–shell nanofibers deliver growth factors and antibiotics for improved bone healing. (**A**): Reproduced with permission from [102], copyright 2016 Elsevier B.V. (**B**): Reproduced with permission from [103], copyright 2020 American Chemical Society.

Another research described the development of an ingenious pH-responsive drug nanocarrier for controlled release of antineoplastic therapeutics. This intelligent system utilized mesoporous silica nanoparticles (MCM-41) as the cargo container and incorporated pH-sensitive gatekeepers constructed from the pH-responsive polymer poly(4-vinylpyridine) (Figure 5A) [102]. The synthesis involved template-assisted sol–gel processes, followed by the attachment of functionalizable groups onto the pore entrances. These groups acted as anchors for the subsequent introduction of the polymeric gatekeepers via precipitation polymerization. Subsequent studies demonstrated pH-dependent release kinetics for the encapsulated antineoplastic drug methotrexate (MTX). A higher release rate was observed in acidic environments (100% at pH 4, 65% at pH 5, and 17% at pH 7) due to the pH-responsive transition of the gatekeepers from a closed to an open state. This design holds promise for targeted delivery of anti-cancer drugs within the acidic tumor microenvironment, potentially minimizing side effects on healthy tissues.

Expanding the potential of silica-based materials in bone repair, research explored a novel nano-silicate-reinforced hydrogel (SMH) for combined bone repair and drug delivery [119]. The SMH hydrogel incorporated bio-compatible polymer sodium alginate with montmorillonite nanoparticles and harmine, an immunogen that promotes M2 macrophage differentiation. Compared to existing bone repair approaches, SMH demonstrated superior mechanical stability, fostered M2 macrophage polarization, increased pro-healing cytokine secretion, and induced pro-inflammatory M1 to the more desirable M2 phenotype, as confirmed by analysis of gene expression. In vivo models further demonstrated the effectiveness of SMH, showing an increase in M2 macrophages, reduced inflammation, and enhanced bone formation. These findings suggest that SMH holds promise as a powerful immunomodulatory biomaterial for bone regeneration. This research offers valuable insights for overcoming challenges associated with current bone repair techniques.

Recent advancements in nanoengineering have yielded a remarkable breakthrough in silica-based biomaterials for regenerative medicine, specifically targeting osteoporosis. The researchers meticulously engineered dual-functional membranes capable of delivering both a growth factor (rhBMP, recombinant bone morphology protein-2) and an antibiotic (gentamicin) (Figure 5B) [103]. For growth factor delivery, they employed MSNs as efficient encapsulation platforms. The antibiotic compartment utilized a coaxial electrospinning technique, creating a core–shell nanofiber system with an antibiotic-impregnated outer shell. This innovative design facilitated the sustained release of both therapeutic agents, showcasing superior ontogenetic regeneration capabilities compared to commercially available Bio-Gide membranes. Furthermore, the gentamicin-loaded core–shell nanofibers exhibited potent antibacterial activity against various oral pathogens, achieving a remarkable 7 log reduction in bacterial colony-forming units (CFU) for both *Escherichia coli* and *Staphylococcus aureus*. Overall, this research unveils a highly promising strategy for dual drug delivery in guided tissue regeneration (GTR), exhibiting enhanced performance metrics. 

A novel technique was developed for fabricating hierarchical hydroxyapatite-dendritic mesoporous silica nanoparticle (HA-DMSN) scaffolds specifically designed for bone regeneration [120]. These scaffolds incorporated a unique hierarchical pore structure, featuring both nano-pores (6.4 nm) and micro-pores (5 µm). This design aimed to influence cell adhesion, proliferation, and differentiation, all crucial aspects of bone formation. In vitro assessments confirmed the biocompatibility of the scaffolds and demonstrated enhanced osteogenic potential. Key bone marker genes exhibited dose-dependent upregulation, while increased ALP activity and robust alizarin red staining indicated heightened bone formation. Furthermore, in vivo studies using a rat cranial bone defect model yielded promising results. HA-DMSN scaffolds significantly promoted bone formation. Notably, they induced a remarkable bone volume increase of 1.56 ± 0.45 mm^3^ within just 4 weeks after implantation, highlighting their efficacy in accelerating bone regeneration. This work not only introduced a promising fabrication method but also showcased the potential of these hierarchically structured scaffolds for stimulating bone regeneration. This research significantly contributes to the advancement of nanoengineered biomaterials for regenerative medicine.

A recent study explored the development of multifunctional nanostructures for concurrent bone repair and infection management. This endeavor involved modifying mesoporous calcium silicate (CaMSN) with increased strontium content, resulting in Sr-CaMSN and SrMSN configurations [121]. These bioactive silicate nanostructures retained their mesoporous nature, enabling significant loading and effective delivery of antimicrobial peptides (AMPs). Notably, the strontium incorporation did not negatively impact the fundamental physicochemical and structural properties of the nanostructures. Biological evaluations revealed that SrMSN facilitated the osteogenic differentiation of mesenchymal stromal cells while suppressing osteoclast differentiation. Furthermore, incorporating AMP into SrMSNs demonstrated remarkable efficacy against methicillin-resistant *Staphylococcus aureus* (MRSA) biofilm formation and growth. This innovative approach, featuring controlled release mechanisms from degrading SrMSN, presents a promising avenue for concurrent infection prevention and bone tissue regeneration, highlighting the potential of multifunctional nanostructures for advanced bone regenerative therapies.

Regenerative medicine procedures aim to repair and heal damaged tissues, but their success can be significantly hampered by bacterial infections, particularly those caused by multidrug-resistant (MDR) bacteria. Building upon the potential of multifunctional nanostructures in bone regeneration, researchers actively explored novel approaches to combat the growing challenge of antimicrobial resistance (AMR). This study investigated the effectiveness of engineered silica with gold nanoparticles in enhancing the potency of existing antibiotics [122]. Two nanocarriers, mesoporous silica nanoparticles (MSNs) and gold–silica core–shell mesoporous nanoparticles (Au@MSNs), were developed and loaded with ofloxacin (loading capacities: 62.32 and 42.18 mg/g, respectively) and amoxicillin (Amox) (loading capacities: 122.75 and 140.85 mg/g, respectively). The antibacterial activity of these nanocarriers was investigated against various bacteria, including MRSA and *Pseudomonas aeruginosa*. Notably, both nanocarriers effectively delivered Amox, enabling significant reductions in the antibiotic dose required to treat resistant strains. Amox@MNs and Amox@Au@MNs reduced the amount of amoxicillin needed by 10-fold and 20-fold, respectively, to treat *P. aeruginosa*. Additionally, Amox@MNs achieved a 20-fold reduction against MRSA. This research reinforces the promising potential of nanocarrier technology for several applications. It holds promise for concurrently promoting bone repair and addressing infections and offers valuable insights for developing next-generation antibiotic therapies to combat the broader challenge of antimicrobial resistance.

### 3.2. Engineered Silica-Based Scaffold for Tissue Engineering

The field of tissue engineering stands at the forefront of medical innovation, striving to address the ever-growing demand for solutions to tissue loss and dysfunction. In this pursuit, silica-based materials have become a compelling class of biomaterials, captivating researchers with their unique combination of biocompatibility, adaptable properties, and inherent ability to foster cell growth and differentiation. This section delves into the thrilling potential of silica-based scaffolds for tissue engineering. We will explore their diverse applications and the intricate interplay between their physicochemical properties and their ability to mimic the native tissue microenvironment, ultimately leading to advancements in tissue repair and regeneration.

One area of intense investigation focuses on BMP2, a potent growth factor crucial for bone formation. However, its clinical use faces limitations, including poor adsorption to traditional carriers and low loading efficiency. A recent study addressed these challenges by developing a silica matrix for BMP2 immobilization and sustained delivery [104]. In vitro investigations using MC3T3-E1 pre-osteoblast cells demonstrated the remarkable ability of silica-coprecipitated BMP2 to induce osteogenesis, even at low concentrations that would not normally trigger bone formation on their own. This suggests that the silica matrix may enhance the potency of BMP2. Furthermore, silica encapsulation significantly improved the thermal stability of BMP2 compared to its free form. The study also compared the release profile of BMP2 from the silica matrix to a traditional BMP2/hydroxyapatite (HA) delivery system. While traditional systems exhibit a burst release of BMP2, the novel BMP2 co-precipitated with silica (BMP2@Si/HA) displayed a sustained release profile. This sustained release translated to superior bone regeneration in a rat calvarial defect model. Animals treated with BMP2@Si/HA exhibited significantly improved bone formation compared to those receiving BMP2/HA.

**Figure 6:**
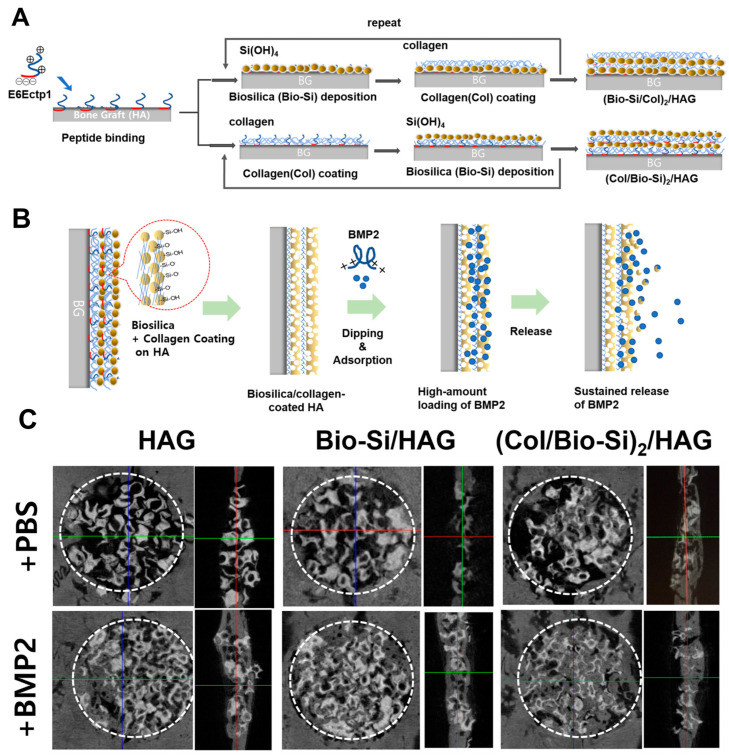
Biomimetic scaffolds for enhanced bone regeneration. (**A**) Schematic of HA core scaffold preparation with collagen layering and Ectp1-mediated silica deposition for various configurations. (**B**) Schematic depicting growth factor (e.g., BMP2) adsorption and release on (Col/Bio-Si)_2_/HA scaffold surface. (**C**) µCT images (transverse and coronal) of rat calvarial bone regeneration after implantation with scaffolds (without or with BMP2). (**A**–**C**): Reproduced with permission from [15], copyright 2023 Elsevier B.V.

Building on the success of silica matrices for BMP2 immobilization, Ki et al. explored an alternative approach to address the limitations of BMP2 in tissue engineering (Figure 6) [15]. Atomic force microscopy revealed the significantly stronger binding affinity of BMP2 to biosilica-coated HA compared to both uncoated and plain silica-coated counterparts. This enhanced affinity contributed to improved loading efficiency of BMP2 onto the carrier material. To achieve controlled release of the bound BMP2, collagen was strategically introduced between the silica layers. This optimized biosilica/collagen formulation facilitated sustained BMP2 release without compromising its initial loading efficiency. In vitro and in vivo experiments using rat calvarial defect models further established the superiority of this novel approach. Compared to traditional methods, the optimized formulation demonstrated enhanced bone regeneration. This study highlights the efficacy of BMP2 affinity carriers for in situ loading and delivery, providing a valuable tool for tissue engineering applications. This innovative strategy has the potential to significantly simplify growth factor utilization through a simple soaking method, thereby facilitating translation to future clinical applications.

Bioprinting revolutionizes tissue engineering by enabling the precise three-dimensional (3D) arrangement of biomaterials, cells, and even bioactive molecules [123]. This innovative technology goes beyond traditional printing by building scaffolds layer-by-layer, offering unmatched control for various biomedical applications. Notably, the inclusion of MSNs within bioprinted constructs can significantly enhance their mechanical properties, a crucial factor for mimicking the strength and support needed for bone regeneration. This enhancement occurs because silica increases the crosslinking density within the scaffold, leading to greater stability and stiffness. Researchers addressed the challenge of temperature-dependent viscosity in gelatin methacrylate (GelMA), a popular biomaterial for 3D bioprinting, by employing a two-stage temperature control system [105]. This approach optimized GelMA’s printability and enabled the fabrication of scaffolds incorporating silane-functionalized silica particles at varying concentrations. The resulting silica composites displayed a significant improvement in mechanical strength, superior cell attachment, proliferation, and osteogenic differentiation compared to control scaffolds. These findings collectively demonstrate the benefits of silica incorporation. It enhances printability, improves mechanical properties through increased crosslinking, and promotes favorable cellular responses in GelMA bioprinted constructs. These advancements support silica’s potential application in hard tissue regeneration.

A recent study explored the specific advantages of incorporating functionalized MSNs loaded with calcium, phosphate, and dexamethasone (MSNCaPDex, with a diameter of 63 ± 8 nm) within bioprinting inks [124]. While emphasizing the advantages of GelMA for bioprinting and cell adhesion, this research introduced MSNCaPDex as an inorganic building block due to its known osteogenic potential. These nanoparticles contributed to a photo-crosslinkable bioink suitable for 3D printing with stem cells. The resulting bioprinted constructs exhibited desirable characteristics, including structural stability, uniform nanoparticle distribution, and apatite deposition, suggesting promising bioactivity for bone regeneration applications. Importantly, human bone-marrow-derived mesenchymal stem cells (hBM-MSCs) seeded within these constructs remained viable and differentiated towards bone-forming cells, demonstrating the bioink’s efficacy in promoting bone repair and regeneration without external stimuli. This innovative approach, combining the strengths of biocompatible GelMA with bioactive MSNCaPDex, presents a promising avenue for further development of effective bioinks for bone tissue engineering applications.

Building on the potential of bioprinting, another research group explored a novel technique combining aqueous polypeptide-based inkjet printing with bioinspired silicification to fabricate functional silica-silk micropatterns [22]. Silaffin-R5, acting as a peptide bioink, was directly written onto a self-assembled silk hydrogel, generating intricate micropatterns [106]. By introducing a silicic acid precursor, the controlled growth of silica particles specifically targeted the silaffin-R5 micropatterns under physiological conditions. This approach allowed for remarkable dimensional flexibility, scaling the micropatterns from micrometer to submeter dimensions, enabling them to align with actual human tissue measurements. Notably, these micropatterned structures exhibited promising potential for directing the orientation of human mesenchymal stem cells (hMSCs) along the micropattern’s direction in vitro. Beyond the showcased application, this biohybrid technology offered a versatile platform for designing micropatterns with diverse materials, including biomacromolecules like polypeptides and growth factors. This versatility had the potential to fulfill unmet needs in biomaterials and regenerative medicine [125]. While the current system exhibits promising biocompatibility, further enhancements are being explored to achieve mechanical properties closer to native tissues. One such approach involves incorporating a hydrophilic, biocompatible macromolecule like BSA into the silk fibrils, prior to silica deposition [126]. This strategy significantly increased the toughness of the silk–BSA hydrogel microfibers and enhanced their energy dissipation within the network. Additionally, the microfiber hydrogel became more resistant to catastrophic failure due to sacrificial interactions between BSA and silk, offering a significant advantage over pure silk microfibers.

A recent study introduced a novel silica-based inorganic–organic hybrid biomaterial for 3D printing as bone substitutes [107]. This biomaterial combined Poly(MMA-co-TMSPMA) star polymer with silica, facilitating the direct ink writing of a sol into scaffolds with pore channels ranging from 100 to 200 µm. These hybrid scaffolds exhibited mechanical properties akin to trabecular bone, with in vitro studies demonstrating the adherence of pre-osteoblast cells regardless of their composition. Notably, scaffolds with a 40:60 inorganic-to-organic composition ratio showcased osteogenic and angiogenic characteristics in a rat calvarial defect model. They promoted the formation of new vascularized bone and induced the polarization of macrophages towards the M2 phenotype. This 3D printing approach with complex polymer structures opens exciting possibilities for the development of advanced bone graft materials.

Addressing the critical need for controlled degradation, another study introduced enzyme-cleavable inorganic–organic hybrid inks for 3D-printed scaffolds [127]. This research introduced the first enzyme-cleavable polymer–silica sol–gel hybrids, specifically designed to mimic natural bone remodeling through degradation in response to endogenous collagenases. These hybrids achieved sustained degradation, exhibiting 10% degradation after two months of enzyme exposure. To investigate the impact of polymer architecture on the hybrid’s properties, researchers synthesized three different star polymers with varying positions of trimethoxysilylpropyl methacrylate (TMSPMA) groups while maintaining consistent overall chemistry and composition. Interestingly, the position of these groups significantly influenced the properties of the final hybrid material, including its degradation rate, mechanical properties, and printability. The star polymer with the TMSPMA inner-star architecture demonstrated the slowest degradation, the most desirable combination of flexibility and toughness, and the most suitable gelation time for 3D printing. This research demonstrates the tunability of enzyme-cleavable and silica-containing hybrids by strategically controlling polymer architecture and TMSPMA group positioning. This approach holds promise for the development of next-generation biomaterials with tailored properties for bone regeneration.

**Figure 7:**
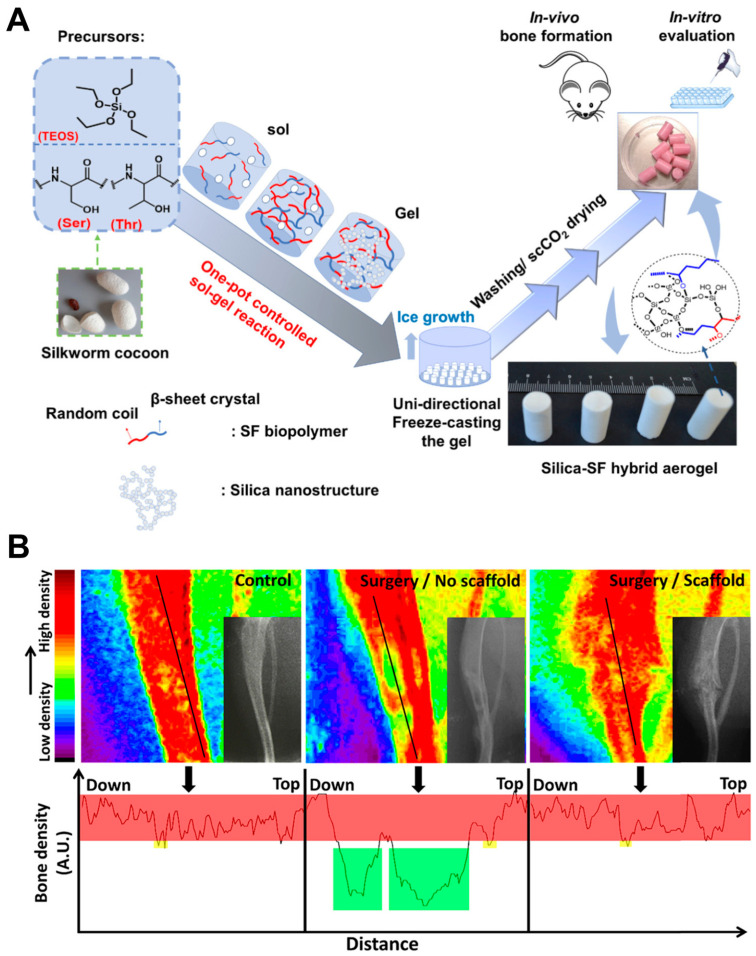
Fabrication and bone formation evaluation of silica–silk fibroin aerogel scaffolds. (**A**) Schematic of silica–silk fibroin (SF) aerogel scaffold synthesis using sol–gel reaction, unidirectional freeze-casting, and supercritical drying. (**B**) Radiographic analysis with color-coded bone density based on radiographs (lower right). Red indicates high bone density, green represents minimal bone formation. Black line marks quantified bone area. (**A**,**B**): Reproduced with permission from [128], copyright 2019 American Chemical Society.

Building on the progress in silica-based materials for bone regeneration, research into silica-based aerogels has yielded promising results [128]. This study presented a novel one-pot synthesis method for silica–silk fibroin hybrid aerogels with precisely controlled pore sizes and superior mechanical strength (Figure 7). These features mimicked the natural structure of bone and provided a suitable environment for bone cell growth and migration. Furthermore, in vitro and in vivo studies confirmed the cytocompatibility, non-hemolytic nature, and ability of these aerogels to promote osteoblast cell growth and new bone tissue formation, showcasing their potential as bioactive and osteoconductive scaffolds for bone regeneration. The cytocompatibility ensured the material’s safety for use in contact with living cells, while the non-hemolytic nature eliminated concerns about disrupting red blood cells. Additionally, the ability to promote osteoblast cell growth and stimulate new bone formation positioned these aerogels as strong candidates for future clinical applications.

Silica–biopolymer hydrogel-based materials are at the forefront of various emerging biomedical applications, particularly in bone tissue engineering [129]. The integration of calcium phosphates within this hybrid network offers a unique strategy for designing implants with captivating biological properties. A recent study presented a method to synthesize silica–chitosan–tricalcium phosphate (TCP) xerogels with varying content of each component. This method utilized a sol–gel process assisted by an ultrasound probe, enabling precise modulation of the material’s properties. The study further investigated the impact of the chosen washing solvent (ethanol or water) on the textural characteristics of the xerogels and successfully confirmed the formation of interpenetrating hybrid structures through Fourier-transform infrared spectroscopy. The study explored how variations in the washing solvent and TCP content influenced the xerogels’ biodegradation, in vitro bioactivity, and osteoconduction. Interestingly, ethanol-washed samples containing calcium and phosphate exhibited the release of both calcium and silicon ions in vitro, while water-washed samples released only silicon. Despite the washing solvent employed, all samples demonstrated in vitro bioactivity and enhanced cell growth in simulated body fluid. Another study focused on further improving scaffolds for bone regeneration by incorporating mangiferin, a bioactive plant compound, into chitosan–silica hybrid nanocomposite scaffolds [130]. Utilizing sol–gel and freeze-drying techniques, they investigated how establishing a 3D crosslinked network and incorporating ZnO nanoparticles affected the physicochemical and mechanical properties of the scaffolds. This approach allows for tailored properties such as porosity, fluid uptake, morphology, thermal stability, and mechanical strength, rendering the scaffolds highly suitable for bone tissue engineering applications. Furthermore, investigations into biomineralization and cell viability have underscored the potential of these hybrid nanocomposite scaffolds, particularly those containing mangiferin, in promoting guided bone regeneration.

**Figure 8:**
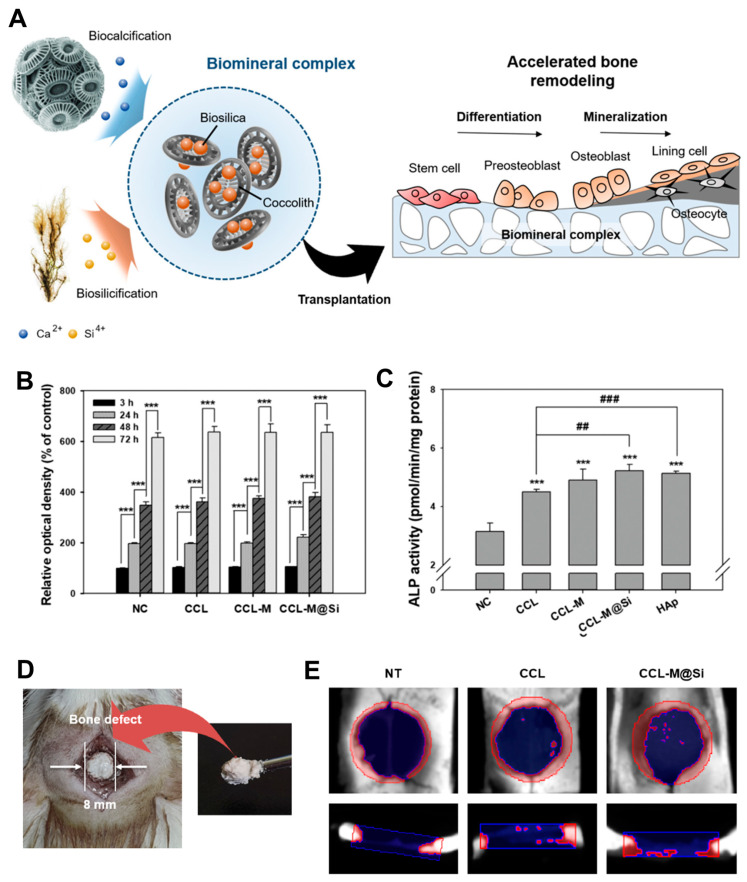
Biomineral complex for bone regeneration. (**A**) Schematic of the complex promoting surface bone formation. Evaluation of mesenchymal stem cell (MSC) behavior. (**B**) Cell proliferation at 72 h. (**C**) ALP activity after 5 days. Significant differences were observed between groups (^##^ *p* < 0.01; ***, ^###^*p* < 0.005). (**D**) Implantation of coccolith-based bone substitute. (**E**) Micro-CT images show bone regeneration over 6 weeks. Newly formed bone in red, defect areas in blue (cross-sectional and sagittal views). (**A**–**E**): Reproduced with permission from [108], copyright 2021 American Chemical Society.

In the quest for innovative bone regeneration solutions, researchers explored a biomimetic approach utilizing a complex derived from marine organisms. This novel biocomplex, comprising biosilicified coccoliths extracted from coccolithophores and bioengineered mussel adhesive protein (MAP) fused with silica-forming peptide (EctP1), holds promise as a viable alternative to traditional inorganic bone graft materials (Figure 8) [108]. The study highlighted the potential of this biomimetic strategy through in vitro and in vivo experiments, demonstrating the biocomplex’s ability to promote bone formation. This beneficial synergy stemmed from the combined effects of osteoconductive calcium carbonate, serving as a scaffold to support bone growth, and osteoinductive silica, which triggered signals within cells to promote their proliferation and differentiation into bone-forming cells. The findings suggest that this biomimetic approach could address limitations associated with existing bone grafts, which often lack inherent osteoinductivity and rely solely on osteoconduction. This study lays a strong foundation for further development of biomimetic strategies in bone tissue engineering. These strategies have the potential to deliver more effective and clinically relevant solutions for bone regeneration.

Collectively, these studies underscore the transformative potential of silica-based materials for advancing bone regeneration strategies. By enabling tailored properties, incorporating bioactive functionalities, and demonstrating superior performance in pre-clinical studies, silica-based hybrids and aerogels hold significant promise for future clinical translation. Their ability to address limitations of existing bone repair methods, such as poor osteoconductivity or weak mechanical properties, and promote new bone formation suggests potential for substantial improvements in patient outcomes and the field of bone tissue engineering.

### 3.3. Engineered Silica-Based Materials for Stem Cell Therapy

The integration of SiNPs into stem-cell-based regenerative medicine offers significant potential for therapeutic innovation in tissue engineering and regenerative applications. These nanoparticles, particularly those incorporating bioactive elements like calcium and phosphate ions, provide a versatile platform to influence stem cell behavior.

A recent study highlighted the development of bioactive SiNPs-CaP, with diameters below 100 nm and containing calcium and phosphate ions, with significant implications for stem cell therapy in bone regeneration [109]. These nanoparticles, synthesized through a sol–gel approach with post-synthesis functionalization for ion incorporation, exhibited a narrow size distribution, good colloidal stability, high ion content, and excellent cytocompatibility. Notably, SiNPs-CaP demonstrated the ability to induce osteoblast proliferation and differentiation, as well as upregulate the expression of bone-related proteins in human bone marrow mesenchymal stem cells (hBMSCs) upon a single-dose administration. This suggests their potential to promote osteogenic differentiation, making them promising candidates as carriers in bone tissue engineering applications, particularly when used in conjunction with stem cell therapy. This development represents a significant advancement in the convergence of nanotechnology and stem cell biology for therapeutic interventions, particularly in promoting stem cell differentiation and enhancing their regenerative properties. The ability to fine-tune the properties of SiNPs, coupled with their versatile functionalities, opens exciting avenues for further research and development in this field, with the potential to revolutionize stem-cell-based therapies for bone repair.

**Figure 9:**
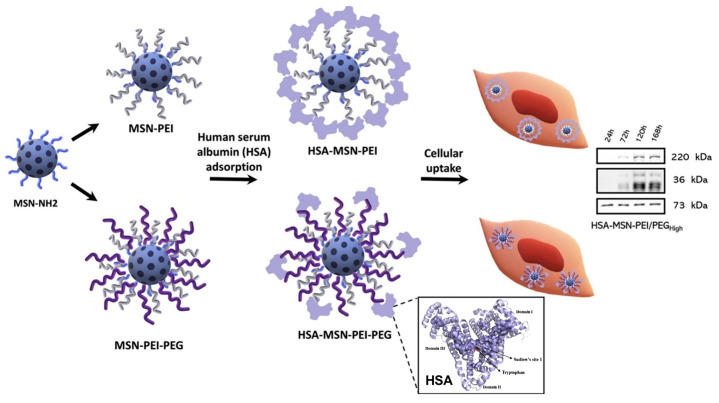
Schematic illustration of the process of human serum albumin (HSA) adsorption and cellular uptake of MSN-PEI nanocomposites by stem cells. Reproduced with permission from [16], copyright 2020 Elsevier B.V.

Silica-based stem cell therapy holds promise for clinical applications, but understanding the behavior of NPs in xenogenic-free stem cell cultures is crucial. Surface modification of MSN, such as human serum albumin (HSA) association, enhanced their compatibility with stem cell therapy. Özliseli et al. explored the impact of HSA on MSNs, a promising material for stem cell therapies (Figure 9) [16]. Their findings demonstrated that HSA coating on MSNs within xenogenic-free media offered several advantages. HSA enhanced the colloidal stability of MSNs, promoting their consistent behavior in cultures. Additionally, they observed variations in HSA adsorption depending on MSN surface modification, with MSN-PEI/PEGHigh leading to protein denaturation. Furthermore, HSA association with MSNs improved stem cell viability without affecting their differentiation potential. Interestingly, the study revealed that MSN internalization by stem cells could be optimized through surface modifications and pre-treatment with HSA. This research paves the way for utilizing HSA-coated MSNs in xenogenic-free stem cell cultures. This approach holds promise for safer and more effective stem cell-based therapies in regenerative medicine applications.

A recent study explored the potential of SiO_2_ to develop injectable bone pastes specifically designed to support stem cell therapy applications in bone regeneration. This research compared two biocomposite hydrogels, silica–nano-hydroxyapatite (SiO_2_/n-HA) and alginate–nano-hydroxyapatite (Alg/n-HA), to evaluate their potential application in bone regeneration [131]. The hydrogels underwent thorough assessment for porosity, viscosity, setting time, mechanical properties, and their impact on mesenchymal stem cell viability. The results revealed distinct advantages for silica compared to alginate. Silica exhibited enhanced biodegradability, facilitating controlled material degradation to support tissue regeneration, and increased water absorption, fostering a favorable microenvironment for stem cell growth and nutrient exchange within the paste. Although alginate-based hydrogels displayed superior mechanical strength, a key factor for initial stability, the unique combination of biodegradability and water absorption offered by silica positions it as a preferable candidate for applications requiring faster tissue remodeling and enhanced cell–material interaction. Notably, both options demonstrated excellent cytocompatibility and significantly contributed to wound healing, solidifying their potential as tools for stem-cell-based bone regeneration.

**Figure 10:**
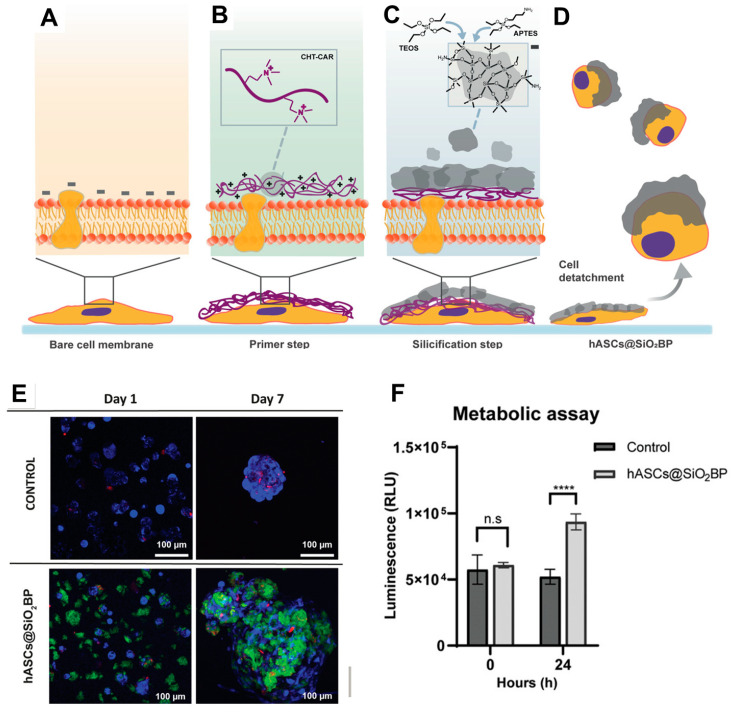
Bioinspired silicification approach for engineering hASC surface. (**A**) Cell membrane bare surface, negatively charged. (**B**) Primer step. Chitosan–carnitine (CHT-CAR) interacts with cell membrane, forming a positively charged layer. (**C**) Biosilicification step and (**D**) detached cells with partial coating, forming hASCs@SiO_2_BP. (**E**) Cell viability assay at days 1 and 7. Live cells (blue), dead cells (red), silica (green). (**F**) Metabolic activity at 0 and 24 h. **** *p* < 0.0001, n.s, not significant. (**A**–**F**): Reproduced with permission from [110], copyright 2021 Wiley-VCH GmbH.

A groundbreaking strategy for enhancing human adipose-derived mesenchymal stem cell (hASC) survival and functionality was achieved through the engineering of silica backpacks onto their surfaces [110]. Inspired by natural biosilicification processes, Maciel et al. developed a novel chitosan (CHT) derivative for a two-step silicification process (Figure 10). Silica backpacks were successfully created on individual hASCs using electrostatic interactions between the CHT derivative and the cell membrane, followed by a biocompatible sol–gel process. Importantly, this approach achieved silica formation without compromising cell viability, unlike methods relying on highly charged polycations. The resulting silica backpacks significantly improved hASC survival in suspension conditions and also promoted a more adherent phenotype upon spreading. This innovative cell-surface engineering technique paves the way for a new generation of hybrid materials with functionalized silica backpacks. Interestingly, future research directions were proposed to explore silica backpack functionalization with specific molecules and investigate the applicability of this protocol to other cell types for differentiation, sensing, and targeted therapies.

Another study explored the potential of 3D-printed scaffolds with controlled channel sizes and silica content to control stem cell differentiation for cartilage repair, emphasizing the critical role of silica in this process [111]. The inclusion of silica in the scaffolds, particularly in conjunction with channels around 230 μm in width, encouraged the formation of hyaline cartilage, as indicated by the prevalence of collagen Type II. This suggested that silica influences signaling pathways that drive chondrogenic differentiation. Conversely, scaffolds with larger channels (~500 µm) resulted in the formation of fibrocartilage, a less desirable tissue type for cartilage repair. This observation underlined the importance of precise control over channel size, potentially mediated by silica’s influence on cell–cell interactions within the scaffold’s microenvironment. The study identified a 200–250 µm channel size as optimal for promoting the desired hyaline cartilage differentiation. This finding underscored the potential for synergistically optimizing both channel size and silica incorporation to create a tailored microenvironment that enhances stem cell therapeutic efficacy in cartilage repair. This research supported the development of silica-containing scaffolds with specific channel designs, offering a promising strategy for promoting cartilage repair and potentially leading to paradigm shifts in treatment approaches for cartilage injuries.

### 3.4. Engineered Silica-Based Materials for Biomedical Imaging

Regenerative medicine aims to heal and restore damaged tissues, often relying on real-time monitoring of the process. Silica-based nanomaterials have emerged as powerful tools in this arena, thanks to their unique properties and versatility in biomedical imaging. Silica materials can be engineered to serve as contrast agents or carriers for imaging probes, enabling real-time monitoring of regenerative processes. This versatility and potential for tailored applications make silica-based nanomaterials a valuable asset in the field of biomedical imaging.

**Figure 11:**
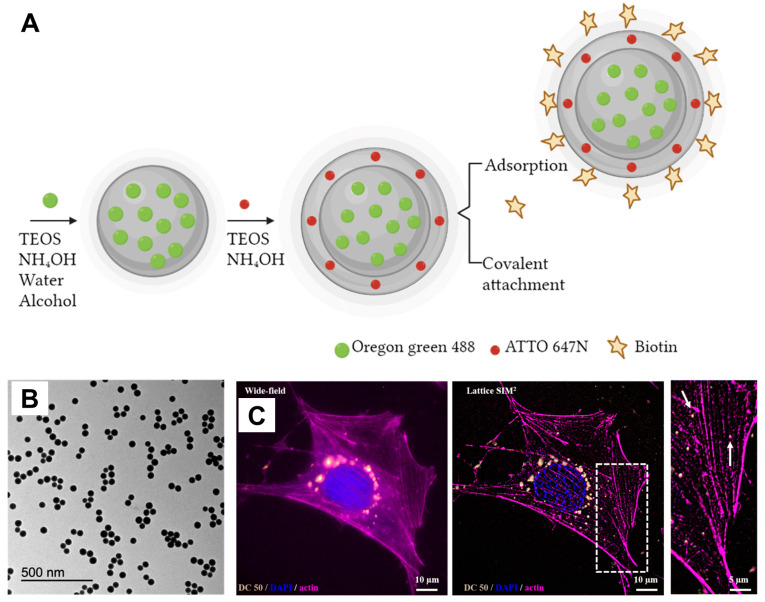
(**A**) Schematic of the two-cycle Stöber method for synthesizing dual-color silica nanoparticles. (**B**) TEM image of silica NPs (49 ± 3 nm). (**C**) Comparison of wide-field and Lattice SIM^2^ imaging of MCF7 cells after incubation with functionalized silica NPs. The inset provides a close-up view of the region marked by the dotted white squares. White arrows highlight features better resolved with Lattice SIM^2^ compared to wide-field imaging. (**A**–**C**): Reproduced with permission from [112], copyright 2023 Royal Society of Chemistry (CC BY 3.0 DEED).

One notable development in this area was the creation of dual-color fluorescent silica nanoparticles (NPs) with potential uses in biomedical applications [112]. This innovative two-step method utilized readily available dyes, Oregon Green 488 and ATTO 647N, for vibrant fluorescence (Figure 11). Additionally, the nanoparticles were functionalized with biotin, enhancing their stability in solution and making them attractive for targeting specific molecules like receptors on cancer cells. The detailed analysis confirmed the nanoparticles’ uniform size and well-defined structure, while advanced microscopy revealed their precise nanometer dimensions, suggesting potential applications in high-resolution fluorescence imaging. The combination of two dyes and biotinylated surfaces further suggested their suitability for targeted cancer cell detection and imaging. This newly developed nanosystem offers promising opportunities for high-resolution fluorescence imaging in regenerative medicine, including the potential for targeted imaging of tissues undergoing regeneration, assessment of therapeutic efficacy, and the detection of disease biomarkers, thereby advancing capabilities in understanding and treating complex biological processes.

Furthermore, the focus on harnessing the potential of MSNs as dual-mode probes for fluorescence imaging and contrast-enhanced magnetic resonance (MR) imaging [113] showcased the importance of silica in advancing biomedical imaging technologies. The incorporation of an aggregation-induced emission (AIE) dye and Gd^3+^ into MSNs through a direct sol–gel method resulted in materials exhibiting strong red fluorescence, making them effective fluorescence probes for microscopy imaging. The addition of Gd^3+^ further enhanced their suitability as contrast agents for MR imaging. The biocompatibility of silica was a key feature highlighted in the study, as demonstrated by a CCK-8 assay, emphasizing its suitability for biomedical applications. The promising performance of these dual-mode probes in both fluorescence cell imaging and MR imaging underscores the significance of silica in advancing biomedical imaging technologies.

Moreover, the utilization of silica in biomedical imaging was further exemplified in the study involving the bio-nanocomplexes for identifying MCF-7 breast cancer cells [114]. The YVO_4_:Eu^3+^@silica-NH-GDA-IgG bio-nanocomplexes were synthesized, incorporating silica to ensure bio-compatibility and conjugation with cancer cells. These complexes exhibited heightened red emission at 618 nm when excited at 355 nm, establishing a strong connection with MCF-7 breast cancer cells through biological conjugation. The findings underscore the potential of silica-based bio-nanocomplexes as a promising labeling agent for applications in biomedical imaging and diagnostics, demonstrating the versatility and significance of silica in advancing diagnostic and imaging technologies in the field of medical research and healthcare.

The development of multifunctional mesoporous silica rods with tailored properties for regenerative medicine applications further highlighted the potential of silica in biomedical engineering [115]. These rods, created through a sol–gel synthesis method, demonstrated capabilities as T2-weighted MRI contrast agents. They were loaded with a cerium compound (in the form of CeO_2_) and functionalized with fluorophores, showcasing the potential for multiple imaging modalities. The in vitro biocompatibility evaluation of the rods in a zebrafish (*Danio rerio*) liver cell line (ZFL) revealed no cytotoxicity for concentrations up to 50 μg/mL, advocating for potential applications in medical imaging and therapy. This study underscores the potential of these multifunctional mesoporous silica rods for diverse biomedical applications, including imaging, targeted drug delivery, and regenerative therapies, due to their biocompatibility and unique shape-dependent properties.

In the field of cancer diagnosis and therapy, there is a growing interest in multi-modality techniques using second near-infrared window fluorescence (NIR-II FL) imaging. A recent development in this area was the FMSN-MnO_2_-BCQ nanosystem, which utilized a degradable silica-based nanoplatform [116]. This innovative system adjusted the intratumoral hydrogen peroxide (H_2_O_2_)/glutathione (GSH) ratio for self-reinforcing CDT and MRI/NIR-II FL imaging. By incorporating BSA-modified FMSN as carriers for a NIR-II small molecule (CQ4T) and the MRI reporter MnO_2_, the nanosystem achieved enhanced biocompatibility, boosted NIR-II fluorescence, and TME-specific degradation. The stepwise degradability of FMSN-MnO_2_-BCQ released MnO_2_ and BCQ nanoparticles in the tumor, enabling the generation of hydroxyl radicals (OH) from endogenous H_2_O_2_ for effective tumor elimination. Additionally, the tetrasulfide bond and MnO_2_ induced GSH depletion, contributing to oxidative cytotoxicity for self-reinforcing CDT. This approach demonstrates great promise in leveraging the potential of silica-based nanomaterials for cancer diagnosis and therapy.

**Figure 12:**
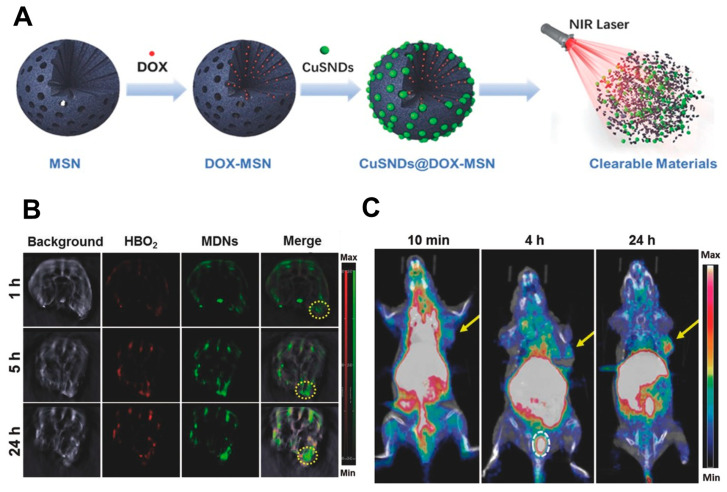
(**A**) Schematic of MDNs formation. In vivo visualization of MDNs post intravenous administration. (**B**) Photoacoustic imaging at different time points, demonstrating selective tumor enhancement (yellow dashed circle). (**C**) PET imaging shows continuous tumor uptake enhancement (yellow arrow), with the bladder area marked (white dashed circle). (**A**–**C**): Reproduced with permission from [117], copyright 2017 WILEY-VCH Verlag GmbH & Co. KGaA, Weinheim.

Furthering the development of silica-based biomedical imaging, Wei et al. presented a novel approach to renal-clearable theranostic nanoparticles (MDNs) for multimodal cancer imaging and combination therapy (Figure 12) [117]. Mesoporous silica nanoparticles were strategically chosen as the core platform due to their biodegradability, renal clearance properties, and ability to serve as a host for additional functionality. A key innovation involved the controlled coating of MSNs with sub-6 nm copper sulfide nanodots (CuS NDs). This design addressed the challenge of low tumor uptake often associated with renal-clearable nanoparticles. The CuS NDs not only facilitated extended blood circulation for enhanced tumor accumulation but also contributed to the MDNs’ bimodal imaging capabilities, specifically PET and photoacoustic imaging. Additionally, the porous structure of MSNs enabled high drug loading capacity, reaching up to 20.2 wt%. Notably, the MDNs exhibited photothermally triggered drug release, leading to synergistic effects in chemo-photothermal therapy. This approach was validated in vivo using two different tumor models. The biodegradable nature of MSNs and the renal-clearable CuS NDs ensured rapid degradation and excretion, minimizing potential long-term toxicity. This research paves the way for a new generation of single-compartment silica-based theranostic agents with integrated multimodal imaging and therapeutic functionalities, holding significant promise for future clinical applications in cancer diagnosis and treatment.

The diverse applications and promising results of silica-based nanomaterials in biomedical imaging underscore the versatility and importance of silica in the development of effective probes with applications in biomedical imaging. These advancements hold significant potential for enhancing diagnostic and imaging technologies in the field of medical research and healthcare.

## 4. Conclusions and Future Outlooks

Engineered silica-based biomaterials are rapidly transforming regenerative medicine. Their unique properties—biocompatibility, tunable porosity, and the ability to interact with cells at the molecular level—make them highly desirable for diverse applications aimed at promoting tissue repair and regeneration. In this review, we explored the foundation of these advancements by examining various approaches for silica synthesis and functionalization. Techniques such as sol–gel processes, including the Stöber method, provide precise control over particle properties (size, morphology, porosity) for tailored functionalities. Moreover, the review emphasized the significance of biomimetic and bioinspired approaches for silica mineralization. By mimicking natural processes of silica formation in organisms like diatoms, researchers could create silica structures closely resembling the intricate architecture of the extracellular matrix (ECM) found in natural tissues. This biomimetic design principle has led to the development of next-generation biomaterials that harmonize with the body and promote superior cellular interactions. Building on this foundation, engineered silica has made significant strides in targeted drug delivery, tissue engineering scaffolds, influencing stem cell behavior for enhanced regeneration, and non-invasive treatment monitoring through biomedical imaging. These advancements hold promise for improved patient outcomes in tissue repair and regeneration.

Significant challenges remain in optimizing engineered silica for regenerative medicine. Precise control over drug release kinetics and material degradation is needed for targeted drug delivery. Long-term biocompatibility requires a deeper understanding of potential immune responses and degradation products. Achieving cost-effective large-scale production through techniques like continuous processing is crucial. Additionally, mimicking the intricate cues of the natural ECM within scaffolds, including both physical structure and biochemical signals, remains a hurdle. Researchers are exploring strategies like incorporating bioactive molecules and compositional gradients. Finally, real-time, in vivo tracking of implanted materials necessitates the development of biocompatible silica that can be visualized using safe imaging techniques like MRI or ultrasound. Overcoming these challenges will unlock the full potential of engineered silica in revolutionizing regenerative medicine.

Despite challenges, the future of engineered silica in regenerative medicine holds immense potential. Advanced functionalization techniques, coupled with state-of-the-art computational modeling and simulation tools, promise the design of next-generation silica-based biomaterials tailored for specific applications. The integration of artificial intelligence (AI) into the design process offers a transformative approach, potentially accelerating the development of highly effective and safe regenerative therapies. Interdisciplinary collaboration between material scientists, biologists, clinicians, and computational modelers is crucial. Bridging expertise across these fields enables a deeper understanding of complex biological interactions and facilitates the translation of discoveries from the lab to the clinical setting, benefiting patients.

The landscape of regenerative medicine is evolving towards personalized treatments, anticipating the use of tailored biocompatible silica materials. Advanced in vivo imaging techniques, coupled with biocompatible silica-based materials which are readily visualized, will enable real-time monitoring of treatment progress and personalized therapeutic interventions. Addressing current challenges and embracing these future perspectives positions engineered silica-based materials as a cornerstone in regenerative medicine, promising breakthroughs in tissue repair and regeneration. Clinical trials and ongoing research provide robust support for the promise of engineered silica biomaterials, contributing to the advancement of a future where these materials routinely contribute to improved patient outcomes.
